# Aerosol Production During Blood and Urine Pre-analytical Processing and Handling in a Hospital Biochemistry Clinical Laboratory During the COVID-19 Pandemic

**DOI:** 10.3389/fpubh.2021.643724

**Published:** 2021-04-21

**Authors:** Marie-Eve Dubuis, Caroline Duchaine

**Affiliations:** ^1^Centre de Recherche de l'Institut Universitaire de Cardiologie et de Pneumologie de Québec, Université Laval, Quebec, QC, Canada; ^2^Faculté des Sciences et de Génie, Département de Biochimie, de Microbiologie et de Bio-informatique, Université Laval, Quebec, QC, Canada

**Keywords:** bioaerosols, SARS-CoV-2, occupational risk, clinical laboratory, blood sample, urine sample

## Abstract

The SARS-CoV-2 pandemic has created a troublesome issue for employees in biochemistry clinical laboratories due to fears of aerosol generation during sample treatment. This study was designed to assess aerosol production during the pre-analytical procedures for blood and urine samples using a model bacterium. Air sampling and surface swabbing were conducted during four typical procedures. Bacteria were not recovered in any air or surface samples. Other studies have reported low and undetectable SARS-CoV-2 RNA in blood and urine samples, respectively. Therefore, the occupational risk for employees appears to be low in terms of aerosol exposure from processing SARS-CoV-2 patient samples.

## Introduction

Clinical biochemistry laboratories (CBLs) treat and analyze hundreds of patient samples on a daily basis. The majority of the samples are from total blood, urine, cerebrospinal fluid, bronchoalveolar fluid and other fluids.

According to the internal procedures at the CBL that was visited, samples are usually treated on a bench without aspiration. Gloves are mandatory for the majority of the procedures. When there is a suspected or confirmed case of certain infectious diseases, employees can prepare samples from infected patients in a biosafety level (BSL) II cabinet and wear additional protective equipment such as procedure masks and safety glasses. Bronchoalveolar fluid samples related to COVID-19 must be treated in a BSL II cabinet.

Since the start of the SARS-CoV-2 pandemic, employees from the visited CBL that process samples from patients who have tested positive have expressed fears verbally to their superiors about the accidental exposure to aerosols during sample pre-analytical procedures. From all the samples treated daily, only a small proportion came from diagnosed or suspected SARS-CoV-2 patients. The virus is still new and these employees have many concerns regarding possible transmission pathways.

Aerosol production during sample treatment and handling in CBLs and other laboratories is recognized by the CDC and the WHO. These procedures include the use of centrifuges, vortex, pipettes, and syringes. Opening sample containers can also produce aerosols if there is a difference in pressure between the container and the room (1, 2). Unfortunately, to the best of our knowledge, quantitative data regarding the generation of aerosol for each type of procedure is not readily available. The aim of this study is to determine whether the main pre-analytical procedures conducted in a CBL produce aerosols.

## Method

### Initial Visit

In order to document potential aerosol production during typical sample processing, our team made an initial visit to a CBL. The treatment of patient samples was observed, particularly for blood and urine samples, as those were more likely to generate aerosols when handled. The visited CBL belongs to the public healthcare system and is a centralized laboratory in a hospital in Quebec City (QC, Canada). Certified laboratory technologists handle all clinical samples. We did not review the internal laboratory's safety and sample management protocols. Human blood and urine samples were not handled during the study, therefore a permission from the Ethics Committee was not needed.

### Description of Blood and Urine Sample Processing

Medical personnel collect blood samples (5 ml) in Vacutainer® Hemogard Lithium Heparin tubes (BD, USA) and send them to the CBL for analysis. The first procedure is to centrifuge each tube in a closed swing-bucket at 4,000 × *g* for 3 min. The centrifugated tubes are then brought to a work space located behind a plexiglass protection panel, and opened. An aliquot of the supernatant is extracted with a disposable transfer pipette (UltiDent Scientific, CANADA) and placed in a small sample container (SSC; Siemens Healthcare Diagnostics Inc., USA) for analysis. Once analyzed, the supernatant is returned to the sample tube using another disposable transfer pipette.

Urine samples (10 to 12 ml) are centrifuged for 15 min at 1,500 × *g*. The supernatant is aspirated using a tube connected to the hospital vacuum system, leaving about 1 mL of the sample in the tube (51.462.901; SARSTEDT AG & Co. KG, GERMANY). The open tube is then vortexed for 20 s before analysis.

### Selection of Aerosol-Generating Procedures

Four common lab procedures that were likely to produce aerosols were selected for analysis: opening blood sample tubes, aliquoting with disposable transfer pipettes, centrifugation of blood sample tubes and vortexing urine samples.

### Selection of a Model Organism

A bacterial culture of *Serratia plymuthica* (ATCC 4261) was used as a surrogate for potential contaminants in blood and urine samples. Colonies of *S. plymuthica* on Peptone Glycerol Agar (PGA) produce a red pigment, prodigiosin (3), which can distinguish them from other environmental airborne bacteria.

### Description of Lab Procedures and Air Sampling

The time required for the experimental procedure and analysis was 6 h: 2 h for the material preparation, 3 h for the air sampling and 1 h for the culture of samples and result readings.

The four lab procedures selected for this research are described below. Three empty blood sample tubes were filled with 5 ml of an overnight liquid culture of *S. plymuthica*. Tryptic Soy Broth (TSB; BD) was also used to fill three other sample tubes, which acted as controls.

Prior to each procedure, the work surface was disinfected with 70% ethanol. Five PGA Petri dishes were opened and placed on the work surface in order to catch aerosolized bacteria that might settle during the experiments.

Air sampling was conducted with an Andersen N6 (Andersen Instruments Inc., USA) coupled with a calibrated high-volume pump (Gast Manufacturing Inc., USA), at a flow rate of 28.3 L/min. The air sampler was placed at a distance of 40–50 cm from the procedure area. Air sampling was initiated 30 s prior to a procedure. Sampling then continued for 2 min during the first and second assays, and 5 or 10 min for the third assay. Air samples for bacteria and TSB were each collected in triplicate. For each procedure, tubes were alternated between replicates during sample collection. There were 5-min wait times after each replicate.

Following the two sets of triplicates (bacteria and TSB), two 10-cm^2^ areas of the work surface were swabbed with flocked swabs (Puritan Medical Products Company LLC, USA). Each swab was stored in a closed tube that contained 1 ml of TSB.

Petri dishes and swabs were stored at 4°C for 2 to 3 h. Tubes that contained the swabs were vortexed and 100 μl of sample liquid was inoculated onto PGA. The inoculations were performed in triplicate. *S. plymuthica* liquid culture was diluted in TSB and then plated on PGA. The Petri dishes were incubated for 48 h at 25°C (first assay) and 72 h at 30°C (second and third assays) before colonies were counted.

Since bacteria pellet at the speed used for centrifugation, for the purpose of this study, the centrifugation step was performed after the first two procedures.

#### Procedure One: Opening Blood Sample Tubes

During air sampling, a tube containing bacteria or TSB was opened, kept open for a duration that was similar to the time taken for sample treatment, and then closed.

#### Procedure Two: Aliquoting Using Disposable Transfer Pipettes

The bacteria and TSB tubes used for the first procedure were opened and kept in a tube rack on the work surface for a wait time of 10 min. Air sampling was initiated and a disposable transfer pipette was used to transfer 100–500 μl of one of the tubes to an SSC. The SSC was installed in another rack, which was brought to the analysis station. After a couple of seconds, the rack was brought back to the work surface and the liquid was transferred into the blood sample tube using another disposable transfer pipette.

#### Procedure Three: Centrifugation

The three TSB tubes were placed in the closed-cap swing-bucket centrifuge and the centrifugation cycle (4,000 × *g*, 3 min) was started. Thirty seconds before the end of the cycle, air sampling was initiated. The centrifuge and the buckets were opened and remained open for the rest of the air sampling period. The three bacteria tubes were then processed as described above.

#### Procedure Four: Vortexing Urine Samples

Air sampling was performed while one sample tube was vortexed for 20 s. Tubes of bacteria and TSB were alternated between each replicate.

### First Assay

The first assay was performed as described above.

### Second Assay

A second assay was performed to validate the air sampling protocol. The fourth procedure was selected for analysis because of its increased potential to produce aerosols. Vortexing is a vigorous process known to generate aerosols. In this instance, opened tubes were vortexed, which could allow the dispersal of the produced aerosols. A 15-ml conical tube was filled with 1 ml of *S. plymuthica* liquid culture or TSB and three PGA Petri dishes were opened and placed on the work surface. Air sampling was performed while a sample tube (bacteria or TSB) was vortexed for 20 s. A 5-min wait time was observed between each replicate. Triplicates were performed for bacteria and TSB.

### Third Assay

The fourth procedure was modified and a third assay was performed. During the longer air sampling periods, the conical tubes were vortexed three times instead of once, punctuated by 30 s wait times. Triplicates for each bacteria and TSB tubes were also performed.

### Calculations

Andersen N6 results were obtained using a positive hole conversion chart (4).

The spray factor (SF) is a ratio that corresponds to the produced aerosol concentration compared to the liquid culture concentration used for the experiments. This factor is used to estimate the significance of laboratory incidents (5).

$$\begin{array}{l} Spray\;Factor=\frac{Aerosol\;Concentration\;\left(CFU/m^{3}\right)}{Liquid\;Suspension\;Concentration\;\left(CFU/ml\right)} \end{array}$$

## Results

### First Assay

After the 48-h incubation period, no colonies were detected on the Petri dishes for air or surface samples, as well as for the liquid culture titration. Samples were incubated for an additional 48-h period to account for possible slow growth, but air and surface samples remained negative. The incubation temperature was then changed.

### Second Assay

After an incubation period of 72 h, no colonies were detected for the air or surface samples. The liquid culture contained 3 × 10^9^ colony forming units (CFU)/ml of *S. plymuthica*.

### Third Assay

The bacterial concentration in the liquid culture was similar to that of the second assay, at 4 × 10^9^ CFU/ml. However, even with the extended air and surface sampling times, *S. plymuthica* colonies were not recovered in the air and surface samples.

### Sample Detection Limit Calculations

Despite negative air and surface samples, we were able to perform some additional analyses. The detection limit can be obtained using the sampling method deployed in this study. Calculations are presented below using sampling times of 5 and 10 min.

The sampled volumes of air were 141.5 and 283 L for 5 and 10 min of sampling, respectively. The detection limit can be calculated using these air volumes and assuming that there is only one CFU per Petri dish. According to the positive hole conversion chart, one CFU corresponds to one particle count.

- 5 min : $\frac{1\;CFU}{141.5\;L}=\;\frac{?}{1000\;L}$, corresponding to 7 CFU per m^3^ of air
- 10 min : $\frac{1\;CFU}{283\;L}=\;\frac{?}{1000\;L}$, corresponding to 4 CFU per m^3^ of air

Consequently, for a 5-min air sampling period with 3 × 20 s of vortexing, <7 CFU/m^3^ of *S. plymuthica* were detected. The detection limit is 4 CFU/m^3^ for a sampling period of 10 min.

### Spray Factor Calculations

The calculated detection limits can then be used to estimate the SF, as described by Dimmick et al. (6, 7).

$$\begin{array}{lll} SF\;5\;min & = & \frac{7\;CFU/m^{3}}{4\;X\;10^{9}\;CFU/ml}=1.75\;X\;10^{-9}ml/m^{3}\\ SF\;10\;min & = & \frac{4\;CFU/m^{3}}{4\;X\;10^{9}\;CFU/ml}=1\;X\;10^{-9}ml/m^{3} \end{array}$$

Therefore, less than two bacteria were aerosolized during the fourth procedure using a liquid suspension of 10^9^ CFU/ml.

## Discussion

The SARS-CoV-2 pandemic raises concerns about employee exposure to the virus when processing infected patient samples. This study was conducted to estimate the potential aerosolization of SARS-CoV-2 from processed blood and urine samples in a CBL. The results obtained using a model organism revealed that for an initial concentration of 10^9^ CFU/ml, the SF is between 1 × 10^−9^ ml/m^3^ and 1.75 × 10^−9^ ml/m^3^. These suggest that sample procedures performed by CBL employees do not produce significant quantities of aerosol. It therefore appears that these employees are unlikely to be exposed to high levels of infectious airborne SARS-CoV-2. However, the number of viruses required to establish an infection, known as the infectious dose, is presently unknown (8). Consequently, there is still some risk when processing samples from infected patients. Differences in the viscosity and content of clinical samples could also influence the behavior of virus aerosolization. When generating aerosols using nebulizers, viscosity of the liquid is inversely proportional to the aerosol size and a high viscosity fluid produces smaller droplets but requires a longer amount of time to nebulize (9).

According to other studies, blood samples from COVID-19 patients can contain SARS-CoV-2 RNA. However, to date, no infectious viruses have been detected in blood samples. A recent article compared viral RNA results from other studies through a systematic review and also conducted a clinical study (10). This systematic review revealed that viral RNA was detected in approximately 10% (95%, CI 5–18%) of the 28 studies that were included, with viral RNA present in 0 to 72% of blood samples. The clinical study revealed 27/212 (12.7%) positive serum samples with RT-PCR cycle threshold (CT) values between 33.5 and 44.8. These high CT values suggest that the genome numbers were relatively low in these samples. The samples that contained detectable RNA were also cultivated on cells, but none led to visible cytopathic effects and there was no increase in subsequent RNA quantification. A small study recovered positive quantifiable blood and urine samples from COVID-19 patients. Between 8.04 × 10^0^ and 9.11 × 10^1^ RNA copies/ml were detected in two blood samples and one urine sample contained 3.22 × 10^2^ RNA copies/ml (11). Another study was able to detect positive quantifiable urine samples in four out of five patients, with concentrations ranging from 1.20 × 10^1^ ± 1.45 × 10^0^ RNA copies/ml to 1.23 × 10^2^ ± 7.08 × 10^0^ RNA copies/ml. In other studies, urine samples contained no quantifiable concentrations of viral RNA (12, 13).

From the highest SF obtained in our study (1.75 × 10^−9^ ml/m^3^) and the highest RNA concentrations found in blood (9.11 × 10^1^ RNA copies/ml) and urine (3.22 × 10^2^ RNA copies/ml), we were able to estimate the SARS-CoV-2 aerosol concentrations that could be generated, which are 1.59 × 10^−7^ RNA copies/m^3^ for blood samples and 5.64 × 10^−7^ RNA copies/m^3^ for urine samples. These estimated potential airborne concentrations remain very low.

One way of reducing aerosol concentrations and protecting employees is to increase the air renewal rate in CBLs. According to the AIA Guidelines for Design and Construction of Hospitals and Health-Care Facilities, there must be a minimum of six air change per hour (ACH) in CBLs (14). New or renovated CBLs can have higher ACH values, therefore limiting exposure to the aerosols that might be produced. There are additional control and mitigation strategies for airborne viruses that could be implemented if resources are available. According to the CDC, precautions can be implemented for specific activities that could generate aerosols or droplets. They include sample handling in a BSL II cabinet or under a splash shield, wearing a face mask or shield and using centrifuge safety cups or sealed rotors. The implementation of such precautions should be evaluated in each CBL depending of the type and the frequency of aerosol-generating activities (15). With the necessary resources and research, hospitals could provide CBL employees with a truly safe working environment.

## Data Availability Statement

The original contributions presented in the study are included in the article/supplementary material, further inquiries can be directed to the corresponding author/s.

## Author Contributions

M-ED wrote the manuscript's draft and contributed in the design of the work. M-ED also acquired, analyzed, and interpreted the data. CD contributed in the design and the interpretation of the work and revised the manuscript. Both authors provided their approval for the publication of the manuscript.

## Conflict of Interest

The authors declare that the research was conducted in the absence of any commercial or financial relationships that could be construed as a potential conflict of interest.
